# Enhanced Electron Emission Performance and Air‐Surface Stability in ScO‐Terminated Diamond for Thermionic Energy Converters

**DOI:** 10.1002/smll.202405408

**Published:** 2024-09-02

**Authors:** Ramiz Zulkharnay, Neil A. Fox, Paul W. May

**Affiliations:** ^1^ School of Chemistry University of Bristol Cantock's Close Bristol BS8 1TS UK; ^2^ School of Physics H.H. Wills Physics Laboratory University of Bristol Tyndall Avenue Bristol BS8 1TL UK

**Keywords:** diamond, negative electron affinity, scandium, surface modification, thermal activation, work function

## Abstract

Diamond with negative electron affinity (NEA) and low work function surfaces are suggested as a suitable material for electron‐generation applications in vacuum, in particular, as the emitter electrode in thermionic energy converters. Such NEA surfaces can be fabricated by evaporating and then annealing submonolayers of a suitable metal in vacuo onto bare or oxidized diamond. Among the metals studied, scandium termination of bare diamond (100) and (111) surfaces is recently reported to give the largest NEA values reported to date for a metal‐diamond system, as well as being thermally stable to 900 °C. It is now shown that preoxidized (100) diamond functionalized with 0.25 monolayers of Sc also produces a large NEA value of −1.02 eV with low work functions (<3.63 eV). Moreover, this surface is thermally stable to 700 °C and can withstand exposure to air for extended periods. Here, the structural and electronic properties of this Sc─O‐functionalized diamond surface are characterized in detail using a variety of surface‐science techniques. The results suggest that this material may be the ideal candidate for the fabrication of commercial thermionic energy conversion devices, e.g., for solar‐power generation, as well as for various other electronic devices that rely upon electron emission.

## Graphical abstract

The Sc─O‐functionalized diamond surface, following thermal activation, exhibits significant resilience against exposure to ambient conditions for extended periods, possessing an NEA of −0.94 eV. These results suggest that this material could be an ideal candidate for the fabrication of commercial thermionic energy conversion devices, such as those used in solar power generation, as well as various other electronic devices that rely on electron emission.

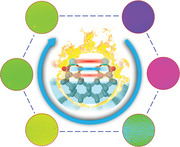

## 1 Introduction

The pursuit of sustainable and efficient energy sources has become increasingly critical in addressing global energy challenges and combating climate change.^[^ ^1^ ^]^ Solar energy, in particular, holds immense potential due to its abundance and renewable nature.^[^ ^2^, ^3^, ^4^ ^]^ Traditional photovoltaic (PV) solar cells have made significant strides in harnessing solar energy, but they are limited by factors such as efficiency losses at high temperatures and low light conditions, etc.^[^ ^5^, ^6^, ^7^ ^]^ To overcome these limitations, alternative approaches, such as thermionic solar‐energy conversion using low‐work‐function cathodes have gained attention for their potential to achieve efficiencies close to the Carnot limit of ≈30–40%.^[^ ^8^, ^9^, ^10^, ^11^, ^12^, ^13^, ^14^ ^]^

Over the past few years, the deposition of diamond via chemical vapor deposition (CVD)^[^ ^15^, ^16^ ^]^ has matured to the extent that relatively inexpensive high‐quality single‐crystal and polycrystalline diamond substrates are now readily available from a number of commercial suppliers for use in a wide range of mechanical, electronic and biomedical applications.^[^ ^17^ ^]^ The availability of such material has enabled detailed studies to be performed on their surface structures, greatly improving the understanding of electron emission from low‐work‐function diamond cathodes. The simplest of these cathodes to produce is hydrogenated diamond because the CVD process uses a predominantly H_2_ atmosphere which automatically terminates the surface with H. However, this type of surface is unsuitable for thermionic energy converter (TEC) devices due to the desorption of H atoms at temperatures above 700 °C leading to a loss of the “negative electron affinity” (NEA). Indeed, simply exposing the H‐terminated diamond surface to ambient air for a few months will partially oxidise it by replacing some of the H's with O atoms and OH groups.^[^ ^18^, ^19^ ^]^

Alternatively, other cathodes utilize bare or oxygenated diamond surfaces terminated with sub‐monolayers of metals such as Li, Mg, Al, Ti, etc. The various termination scenarios aim to create a dipole at the surface which gives rise to NEA.^[^ ^20^, ^21^, ^22^, ^23^, ^24^, ^25^, ^26^ ^]^ The NEA enables electrons residing in the conduction band (CB) to escape into the vacuum without having to overcome a potential barrier. Such thermionic emission is therefore possible with NEA diamond at temperatures ≈500 °C, rather than the 1500–2000 °C normally required for emission from metals.^[^ ^27^ ^]^ Such low‐temperature emission means that NEA diamond is a promising candidate for TECs, which can generate solar power from focussed solar radiation.^[^ ^10^, ^11^, ^12^ ^]^ Nevertheless, practical applications of thermionic solar‐energy conversion require addressing challenges related to electron‐emission efficiency and surface stability.^[^ ^11^, ^17^ ^]^ Thus, the search for a suitable diamond surface termination arises, aiming to achieve high electron‐emission performance at relatively low temperatures (≈500–700 °C), withstand prolonged usage under thermionic emission conditions and/or under ambient conditions in air, and compatibility with commercial TECs currently used for solar power generation.

The best NEA and electron emission results on diamond are typically observed from small, highly charged metallic adsorbates which form strong bonds with C and/or O.^[^ ^11^ ^]^ Reports suggest that the M─O─C_d_ system (where C_d_ denotes a carbon atom on the diamond surface) is generally more air‐stable than the M─C_d_ system. This may be due to the fact that the metal is already partially oxidized which reduces its affinity for further reaction with ambient air. However, for the M─O─C_d_ system, various bonding scenarios between oxygen and surface carbon atoms are possible, including ketone (C_d_═O), bridging ether (C_d_─O─C_d_), hydroxyl (C_d_─OH) or more complex structures such as carboxylic acid groups or lactones.^[^ ^28^, ^29^ ^]^ While these scenarios may be present in varying proportions on the oxidized surface, the challenge of controlling their relative ratios can make the M─O─C_d_ system particularly complex.^[^ ^25^, ^30^ ^]^ Specifically, this complexity arises from the preferential bonding of some metals for O atoms, resulting in a mixed surface coverage of M─O and M─C_d_ bonds, displaying NEA and positive electron affinity (PEA) simultaneously.^[^ ^25^, ^30^ ^]^

To address this, we recently reported a density‐functional theory (DFT) atomistic study on the Sc─O─C_d_ system, identifying the optimal bonding arrangement of scandium with oxygen on the undoped diamond (100) surface and calculating the surface electronic structure, including electron affinity (EA) and work function values, as well as surface stability.^[^ ^31^ ^]^ While the calculated electronic values showed significant dependence on the surface coverage and positioning of the Sc adsorbates, the adsorption of 0.25 monolayers (ML) of Sc onto the oxidized surface was thermodynamically favorable, with a high adsorption energy of −8.68 eV per adsorbate atom, suggesting a thermally stable surface beyond 1000 °C. More significantly, the EA and work function values were computed to be −3.73 and 1.43 eV, respectively, surpassing those of any M─O─C_d_ systems studied to date.^[^ ^32^, ^33^, ^34^, ^35^, ^36^ ^]^

From these results, it appears that ScO‐diamond is a highly promising candidate for a thermionic cathode because Sc meets all the preferred criteria, mentioned earlier, for an adsorbate atom. It is perhaps, unsurprising that Sc appears to be the ideal metallic adsorbate for this application. Reports over the past decade have suggested that the presence of Sc in thermionic cathodes, though not directly acting as an adsorbed cation, contributes to the binding and stabilization of the surface; this mitigates desorption, therefore enhancing work function reduction and/or extending cathode lifespan.^[^ ^37^ ^]^

In this report, we present the first detailed, systematic study of the electron‐emission properties of the ScO‐terminated single‐crystal diamond (SCD) (100) system. Using a simple thermal activation process, the generation of an NEA surface resulting in a high electron‐emission yield, is demonstrated. Additionally, the surface survives thermal annealing up to 700 °C without degradation and remains stable during exposure to ambient conditions. State‐of‐the‐art surface‐science techniques and complementary ab initio DFT calculations were employed to study the changes in the surface electronic states as a function of the activation procedure. This study unveils a pathway toward exploring new avenues in the fabrication of diamond‐based thermionic cathodes for commercial TEC devices.

## 2 Results and Discussion

### 2.1 Surface Structure

These studies generated a large amount of data, so only the results necessary to explain the findings and provide a cohesive narrative are given in this paper. More detailed complementary results can be found in the accompanying Supporting Information. Figures and Tables in Supporting Information are labeled with an “S” prefix, e.g., Figure S1 (Supporting Information).

Experimental spot‐profile analysis low‐energy electron‐diffraction (SPA‐LEED) data from diamond structures, supported by *LEEDpat4* software simulations and DFT calculations were used to elucidate the atomic configuration and surface reconstruction – in real and reciprocal space – before and after Sc addition onto the oxidized SCD (100) surface. An oxidized diamond (100) sample was examined first in order to set a benchmark for the subsequent Sc─O‐terminated structures. The oxygenated diamond surface features a single 90°‐rotated domain in a square lattice, displaying a characteristic (1 × 1) LEED pattern, as shown in Figure S1a (see Supporting Information), and these findings are in good agreement with the LEED results from previous studies.^[^ ^29^, ^38^, ^39^ ^]^

**Figure 1:**
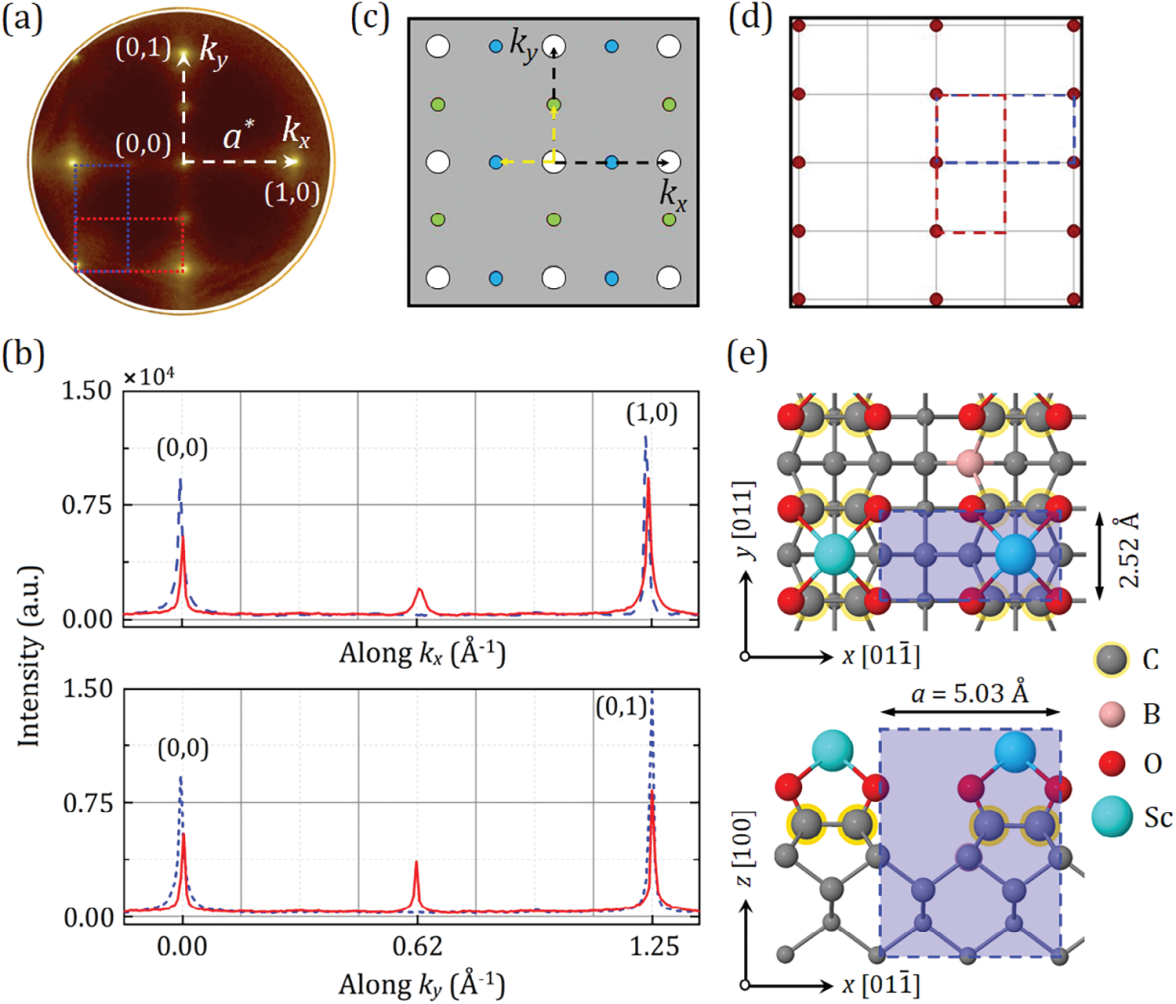
Real‐space and reciprocal‐space patterns of the 0.25 ML Sc‐adsorbed oxidized SCD (100) surface at room temperature. a) Experimental LEED pattern of the ScO‐diamond sample scanned at a beam energy of 130 eV. White dashed arrows indicate the primitive reciprocal‐lattice vectors along the *k_x_* and *k_y_* axes, while blue and red dotted rectangles correspond to two commensurate (2 × 1) and (1 × 2) reconstructions. b) Spot‐profile analysis plots before (blue dashed line) and after (red solid line) Sc deposition along the *k_x_* and *k_y_* vectors. The signal intensity of the diffraction spots is taken with respect to the background. c,d) Simulated superpositions of reciprocal‐space and real‐space patterns for the 0.25 ML Sc‐adsorbed O‐terminated (100) surface, respectively. In panel (c), blue and green circles in a square lattice are shown for O and Sc atoms, respectively, while white circles correspond to the (1 × 1) spots in a square lattice. Black and yellow dashed arrows represent the primitive reciprocal‐lattice vectors indicating (1 × 1) diffraction spots and (2 × 1) or (1 × 2) reconstructions, respectively. In panel (d), the red circles refer to the position of individual domains, while the blue and red dashed rectangles refer to the (2 × 1) or (1 × 2) geometry, respectively. e) Top (above) and side (below) views of the lowest energy structure for the 0.25 ML Sc‐adsorbed O‐terminated (100) surface, modelled by DFT calculations. The surface primitive unit cell is indicated by the blue dashed rectangle, indicating the (2 × 1) or (1 × 2) reconstructions.

In contrast, the deposition of Sc on the oxidized diamond (100) surface resulted in a significant alteration of the surface structure, evidenced by the formation of a (2 × 1) reconstruction, as expected (**Figure** **1**). The reciprocal lattice pattern revealed a double domain within a fourfold rotational symmetry, where the central (0,0) spot was separated by ≈1.25 Å^−1^ from (1,0) along the reciprocal lattice vector *k_x_* (or (0,1) along *k_y_*),^[^ ^40^ ^]^ as illustrated in Figure 1a,b. This spacing value of the reciprocal‐lattice mesh corresponds to a real‐space separation of 5.02 ± 0.02 Å, calculated using the following equation:

(1) $$a=\frac{2\pi }{a^{*}}$$

where *a* and a^*^ are, respectively, the real and reciprocal‐space lattice parameters of diamond. It is worth noting that the central (0,0) spot closely matches (≈99%) the center of the Brillouin zone (Г) for the (2 × 1) reconstructed ScO‐diamond (100) surface, as seen in the LEED pattern for bare diamond C_d_(2 × 1)‐(100).^[^ ^41^ ^]^ Furthermore, the experimentally measured lattice constant of 5.02 ± 0.02 Å is consistent with the DFT‐predicted value (5.03 Å) for the ScO‐terminated boron‐doped diamond (BDD) (100) surface structure, as illustrated in Figure 1e, highlighting the accuracy of DFT modeling in this case.

Figure 1a shows that the integral‐order domains, indicating the (1 × 1) pattern, remain prominently visible and intense. Additionally, the LEED pattern confirms the presence of a faint (2 × 1) reconstruction as inner spots interspersed among the (1 × 1) domains. Although the LEED pattern of the O‐terminated SCD (100) is clear and intense (Figure S1a, Supporting Information), the (2 × 1) surface reconstruction resulting from the adsorption of 0.25 ML Sc is relatively weaker in some inner spots between the first‐order diffraction domains, as evidenced by spot‐profile analysis of the oxidized and ScO‐terminated surfaces (Figure 1b). This can be attributed to the small concentration of scandium atoms compared to a full‐ML coverage of oxygen atoms. Nevertheless, it still provides strong evidence for the occurrence of the ScO termination on the SCD (100) surface.

The experimental findings of the SPA‐LEED study were verified through *LEEDpat* simulations, utilizing a matrix $\left(M=[\begin{array}{cc} 2 & 0\\ 0 & 1 \end{array}]\right)$ consisting of the two domains rotated by 90° on a square lattice, as shown in Figure 1c. This simulated reciprocal‐space pattern is in excellent agreement with a commensurate surface reconstruction of (2 × 1) or (1 × 2) in a real‐space lattice for the 0.25 ML Sc‐adsorbed O‐terminated diamond (100) surface (Figure 1d). The results of the SPA‐LEED analysis provide a strong basis upon which to continue investigating the adsorption of Sc onto the oxidized surface experimentally, particularly regarding the surface electronic structure.

### 2.2 Surface Chemical Composition

**Figure 2:**
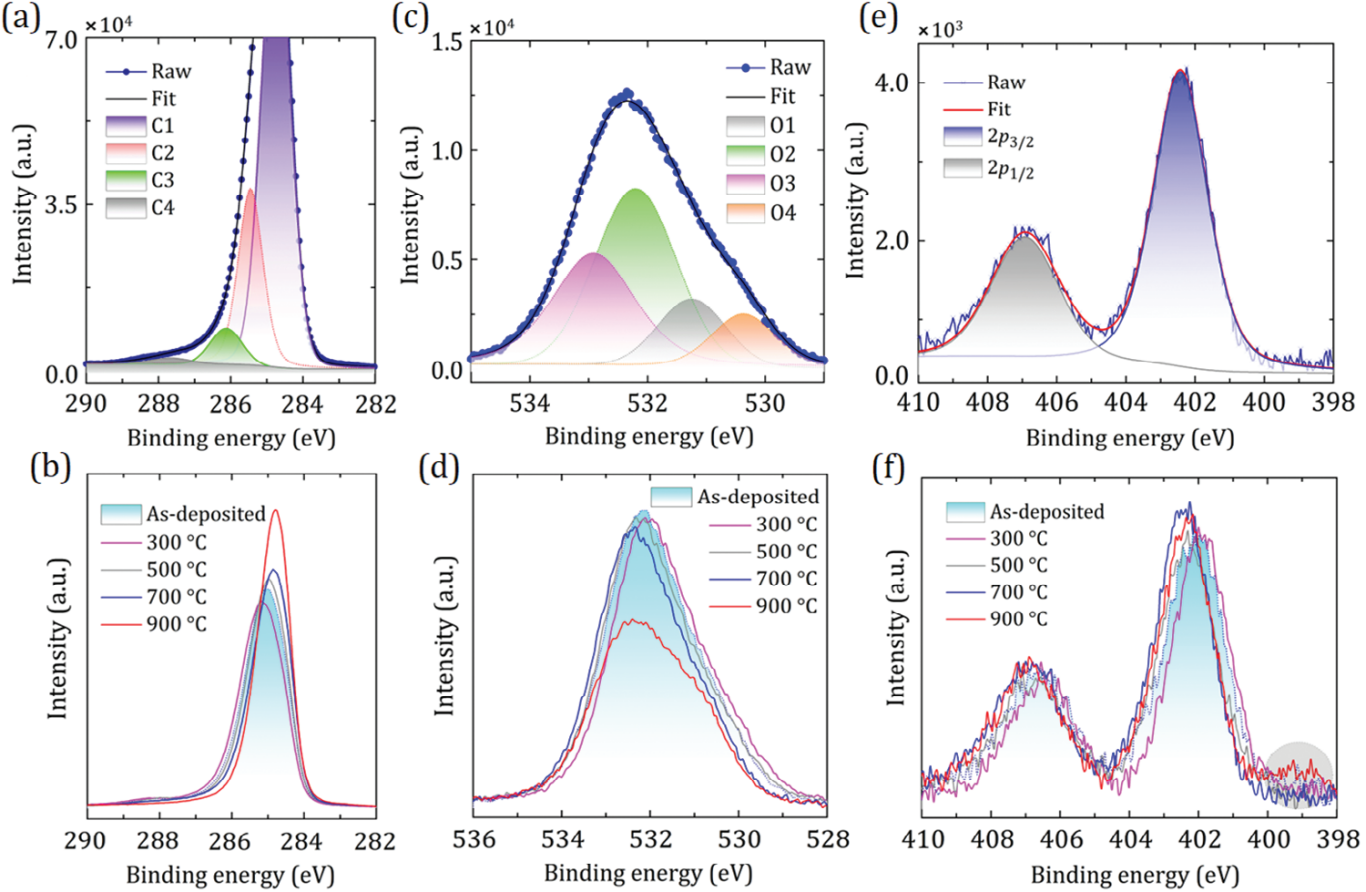
Core‐level XPS spectra of the ScO‐terminated diamond observed at various steps of the sample preparation. The top panels, denoted as (a), (c), and (e), display deconvoluted peaks for C 1*s*, O 1*s*, and Sc 2*p*, respectively, after in vacuo annealing at 700 °C. These panels are extracted from a series of step‐by‐step observations, which are depicted in the lower panels (b), (d), and (f). “As‐deposited” means 0.25 ML Sc deposition at room temperature. In panel (a), the top of the C 1*s* peak is cut off to highlight low‐intensity components. In panel (f), the grey circular region indicates the appearance of the ScC peak with a binding energy of 399.15 eV following the anneal at 900 °C.

To observe chemical changes in the C, O, and Sc core‐level peaks, we now focus on characterizing the elemental composition through core‐level photoemission spectroscopy. Step‐by‐step X‐ray photoelectron spectroscopy (XPS) acquisitions were performed in situ before and after the Sc deposition and following annealing points (**Figure** **2**).

Figure 2a depicts the deconvoluted C 1*s* spectrum resulting from the annealing process performed at 700 °C for 15 min, while a series of C 1*s* peaks obtained at various stages of the sample preparation are presented in Figure 2b. The addition of Sc and subsequent annealing at temperatures up to 900 °C results in a shift of the C 1*s* peak to lower binding energies (BEs), except when annealed at 300 °C (Figure 2b). This chemical shift is consistent with the change in surface dipole, as previously reported.^[^ ^39^ ^]^ The XPS spectrum of the ScO‐diamond surface exhibits similarities to that of the O‐terminated diamond, as shown in Figure S2a (Supporting Information), but with only a negligible presence of surface *sp* ^2^ C atoms. This suggests that no graphitization occurred during the annealing steps, except for a slight increase observed at 900 °C. While the intensities of the C 1*s* peaks remained relatively stable throughout different stages of sample preparation, a significant increase of 5.3% in the intensity was observed after annealing at 900 °C compared to that from the lower‐temperature anneals. This increase in the C peak was due to the desorption of oxygen from the surface which has been observed to occur at temperatures >800 °C.^[^ ^42^ ^]^ This surface composition change is also reflected in an increase in work function for the 900 °C sample, resulting in a slight positive EA compared to that from a sample annealed at 700 °C, as discussed in further detail in section 2.3.

There are four contributions to the C 1*s* peak, as shown in an example from the sample annealed at 700 °C, labeled C1 to C4, presented in Figure 2a. Component C1 centered at 284.85 eV is attributed to the bulk *sp* ^3^ C atoms and is unaffected by the surface termination, consistent with the Li─O─C_d_ structure described by O'Donnell et al.^[^ ^23^ ^]^ Sc adsorption onto the O‐terminated diamond leads to the emergence of an additional peak (C2) at 285.58 eV, corresponding to the Sc─O─C_d_ component. The last two components (C3 and C4) are attributed to C_d_─O and C_d_═O bonds with BEs of 286.56 and 287.82 eV, respectively. Compared to the oxidized diamond surface prepared via the UV‐ozone method, these two components (C_d_─O and C_d_═O) have shifted to lower BEs at 0.12 and 0.44 eV, respectively, upon 0.25 ML Sc deposition and following the annealing up to 700 °С. Additionally, the concentrations of C_d_─O and C_d_═O decreased by 24% and 68% after Sc deposition, respectively, suggesting that, experimentally, Sc adsorption occurs predominantly on the ketone oxygen. This is in excellent agreement with our previous DFT predictions on the ScO‐terminated undoped diamond.^[^ ^31^ ^]^

The deconvoluted O 1*s* spectrum after the anneal at 700 °С is displayed in Figure 2c, along with a set of peaks corresponding to various stages of the sample preparation in Figure 2d. Following the adsorption of Sc and subsequent annealing (excluding the one at 300 °С), the O 1*s* spectral features showed a shift toward higher BEs of up to ≈0.4 eV, indicating a modification of the surface states (Figure 2d). While the O‐terminated surface created with the UV‐ozone method displayed three well‐defined spectral components, C_d_═O, C_d_─OH and C_d_─O─C_d_ (O1, O2 , and O3, respectively), in the O 1*s* spectrum (Figure S2b, Supporting Information), the addition of Sc results in the appearance of a new peak (O4) at a lower BE of 530.37 eV belonging to the Sc─O─C_d_ structure, in good agreement with findings from previous M─O─C_d_ structures.^[^ ^24^, ^25^, ^30^ ^]^ With the deposition of scandium onto an oxidized surface, typically one of the surface bonds, either a π‐bond in the ketone (C_d_═O) or a σ‐bond in the ether (C_d_─O─C_d_), is broken, leading to the abstraction of oxygen atoms from the surface. Moreover, a decrease in the intensity of the O 1*s* peak by 25.4% provides empirical evidence that 0.25 ML of Sc has adsorbed onto the oxidized surface, as expected.

Notably, the intensity of the C_d_═O component (O1) is substantially reduced by 70% following the deposition and further anneal at 700 °C compared to that of the oxidized diamond surface (see Figure S2b, Supporting Information). This reduction is consistent with the decrease in the intensity of C_d_═O (68%) from the C 1*s* peak, as mentioned earlier. Conversely, the intensities of the other two groups (C_d_─OH and C_d_─O─C_d_) are increased by 15% and 27%, respectively, providing further evidence that Sc adsorption occurs preferentially on the ketone‐bonded O‐terminated surface.^[^ ^31^ ^]^

Figure 2d shows that the concentration of O on the surface was 7.56% (equivalent to a full ML) following Sc deposition and 7.38% after annealing at 700 °C. However, two oxygen‐loss events were observed during successive annealing stages at 300 °C (1.59% of the total amount of O atoms) and at 900 °C (26.3%). The first event is due to the desorption of weakly bonded oxygen‐containing adsorbates from the diamond surface at lower annealing temperatures, as previously observed in Li─O─C_d_^[^ ^23^ ^]^ and Mg─O─C_d_^[^ ^24^ ^]^ systems. This also explains the observed reversal of the shift in the C 1*s* and O 1*s* peaks after annealing at 300 °C (recall Figure 2b–d). The second event is believed to result from the thermal release of unbonded oxygen atoms from the surface, as previously reported by Bobrov et al., following temperature‐programmed‐desorption studies.^[^ ^43^ ^]^

The subsequent experiment in which an annealed ScO‐diamond surface was exposed to air under ambient conditions for two weeks showed that the loss of oxygen had been compensated without a significant change in the electronic structure, but it resulted in a slight reduction in work function and EA values (see section 2.3). XPS results of the C 1*s*, O 1*s*, and Sc 2*p* peaks for air exposure are provided in Figure S4 (Supporting Information).

A core‐level spectra analysis of Sc 2*p* in vacuo annealing at 700 °C and a series of spectra at various steps of the sample preparation process are presented in Figure 2e,f, respectively. A similar trend to those of the Sc‐adsorbed bare SCD (100) and (111) samples is observed in the case of Sc 2*p* with successive annealing steps (excluding 300 °C). This trend is characterized by a shift in chemical states that is in the opposite direction to that seen for the C 1*s* peaks, suggesting an increase in the surface dipole density.^[^ ^23^, ^24^, ^39^ ^]^ As shown in Figure 2e, the Sc 2*p* core‐level spectrum consists of Sc 2*p*_3/2_ (402.48 eV) and Sc 2*p*_1/2_ (407.08 eV) peaks with a spin‐orbit splitting of 4.6 eV, in excellent agreement with the literature reports.^[^ ^44^ ^]^

The stability of the Sc concentration through the range of annealing temperatures, with only a slight decrease of 0.6%, supports the conclusion that Sc atoms are strongly bonded to the O‐terminated surface. Post‐annealing at 900 °C, an extra peak appears at a lower BE of 399.15 eV due to a significant decrease in oxygen (Figure 2f). This peak is typically attributed to ScC, suggesting that some of the Sc atoms become bonded to the bare diamond surface following the thermal desorption of O atoms. Therefore, this change in the Sc 2*p* core‐level spectrum highlights the importance of the annealing temperature on the bonding of the Sc atoms to the diamond surface. We conclude that the loss of a fraction of surface adsorbates or termination causes alterations in the surface states, particularly in the surface dipole density.

### 2.3 Electronic Structure

Electronic structure examination of the ScO‐terminated diamond (100) was carried out following each step of the sample preparation, utilizing ultraviolet‐photoelectron spectroscopy (UPS) and energy‐filtered photoemission electron microscopy (EF‐PEEM) techniques. As before, an O‐terminated (100) sample was first studied as a benchmark (Figure S3a,b, Supporting Information, for the full‐scale UPS spectrum and PEEM map of the UV‐ozone treated surface, respectively), and the results were compared with those from the literature.^[^ ^25^, ^29^, ^45^, ^46^, ^47^ ^]^

**Figure 3:**
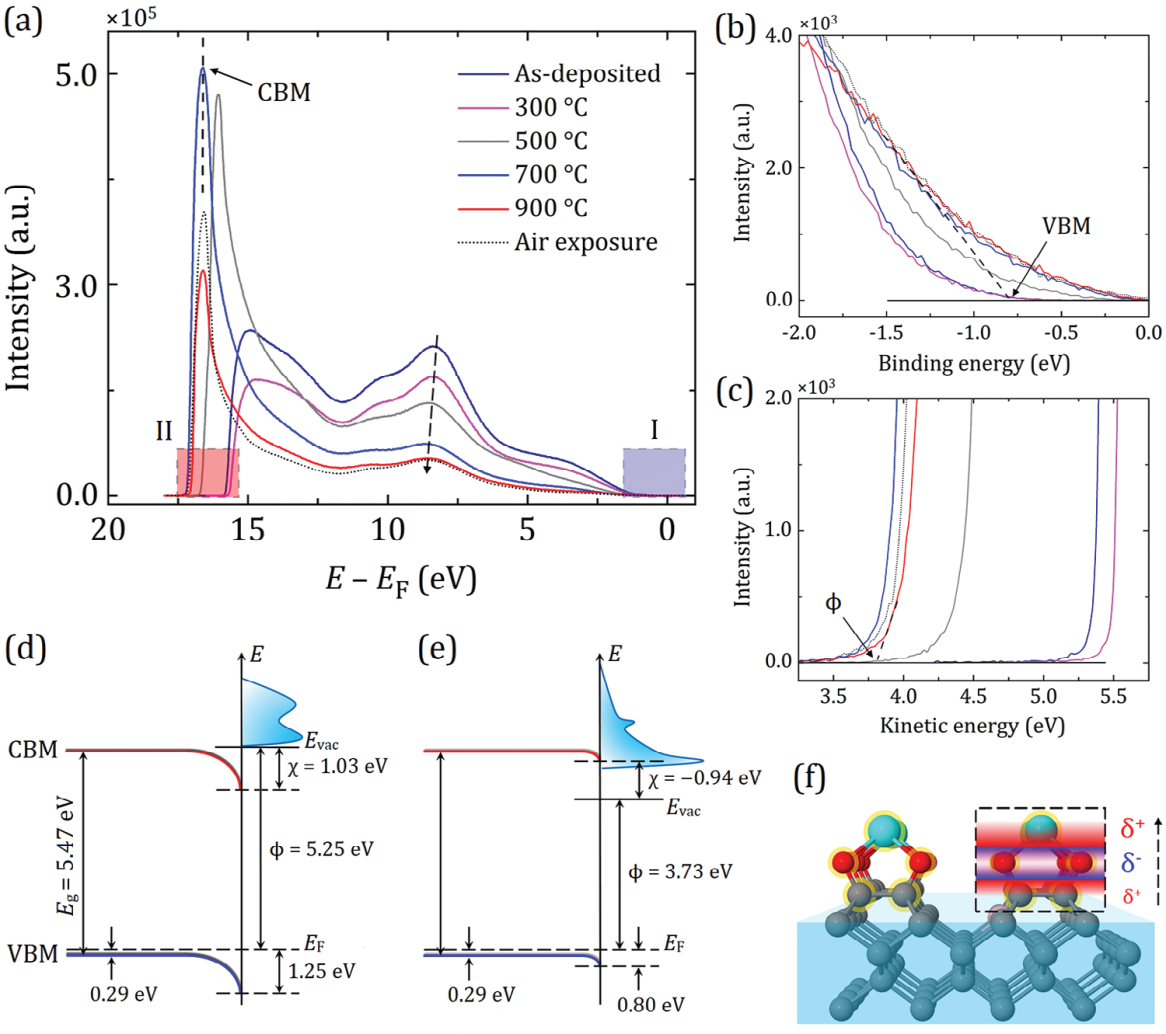
The electronic surface structure of the ScO‐terminated diamond (100). *E*_g_  = bandgap, *E*_F_  =  Fermi level, *E*_vac_  =  Vacuum energy level, ϕ  =  work function, χ  =  electron affinity. a) The full‐scale UPS spectra at various stages of sample preparation (e.g., after 0.25 ML Sc deposition and in vacuo annealing stages up to 900 °C followed by air exposure), labeled with two regions of interest. The regions labeled I and II are defined with red and blue rectangles, respectively, corresponding to the magnified panels (b) the cut‐off energy and (c) the VBM position relative to the Fermi level, respectively. In panel (a), the position of the CBM is indicated by a vertical dashed line at 16.57 eV, in good agreement with the maximum of the secondary‐electron peak, whereas the black dashed arrow serves as a visual guide for the intensity decrease and BE shift. (d) and (e) are energy‐level diagrams for the ScO‐terminated diamond samples, including those as‐deposited and those exposed to ambient conditions, respectively. “As‐deposited” means 0.25 ML Sc deposition at room temperature. f) A schematic illustration of a dipole model for the 0.25 ML Sc adsorbed on the oxidized diamond (100) surface, displaying the outward‐facing dipoles as indicated by the black dashed arrow. The red and blue areas in the dashed square represent the positively and negatively charged regions, respectively.

**Figure** **3** presents the electron density of states (DOS) for the ScO‐diamond surface at each step of the sample preparation, along with the expected energy‐level diagrams for the two most significant stages (i.e., following Sc deposition and air exposure). The full‐scale UPS spectra are shown in Figure 3a, depicting the energy distribution of inelastically scattered electrons (i.e., secondary electrons) for the ScO‐termination. The spectra for the occupied and unoccupied states are divided into two regions of interest, denoted as I and II. The lower energy “region I” mainly comprises the band structure of the occupied states (i.e., the valence band (VB)). Here, the lower‐energy cut‐off in the spectra is determined by pinning the Fermi level (*E*_F_) at a BE of 0 eV, as a reference for all UPS measurements.^[^ ^48^ ^]^ This allows the position of the valence band maximum (VBM) to be discerned by linear extrapolation, as shown in Figure 3b. In contrast, “region II” principally reflects the unoccupied CB states at higher energy, which are populated only following photoexcitation and relaxation by secondary electrons through barrierless emission into the vacuum. This edge of the UPS spectra is no less significant as the difference between the energy of the photon source (*hν*  =  21.22 eV) and the high‐energy cut‐off is assumed to be the work function of the surface of interest (Figure 3c). Here, we note that the secondary‐electron region (Figure 3c) is plotted as a function of “corrected kinetic energy”, as suggested elsewhere.^[^ ^49^ ^]^ This approach allows the reader to directly extract the work function value without needing to reference additional information.

As shown in Figure 3b,c, these close‐up views highlight details that are concealed in the full spectrum display by the dominance of the secondary electron (SE) tail (i.e., the NEA peak), which has a significantly higher intensity compared to the rest of the spectrum. The significant rise in peak intensity during successive annealing steps led to a broadening of the secondary‐electron background toward higher energy levels, ultimately pinning it just above the CBM for the “thermally activated” surfaces (Figure 3a). Significantly, the SE tail further spanned up to 3.63 eV for the most negative EA surface (i.e., the 700 °C annealing), decreasing exponentially in intensity, indicative of a lowering of the work function and a more negative EA, as confirmed by the change in chemical states in XPS results (see section 2.2). In contrast, the O 2*p* VB states at 8.5 eV, accompanied by a shoulder peak ≈10.2 eV, exhibited a reduction in intensity and a slight shift toward higher BEs (up to ≈0.4 eV) as annealing temperatures increase, as indicated by the black dashed arrow in Figure 3a. This is consistent with the thermalization of excited electrons in the CB according to the Maxwell‐Boltzmann distribution.^[^ ^50^ ^]^

Conversely, post‐deposition annealing at 300 °C resulted in lower cut‐off energy compared to that after Sc deposition at room temperature, as confirmed by the work function and EA values. This outcome also confirms the conclusions drawn from core‐level spectroscopy earlier (recall section 2.2). Moreover, the final annealing point of 900 °C did not display the most negative EA value, as seen for the Sc‐terminated bare diamond,^[^ ^41^ ^]^ which is likely due to the significant loss of O atoms from the surface. In fact, the most negative EA value was observed after annealing at 700 °C, with a value of −1.02 eV, and its corresponding work function of 3.63 eV, as listed in **Table** **1**. These values are higher than those reported from the Li─O─C_d_^[^ ^23^ ^]^ and Mg─O─C_d_^[^ ^24^ ^]^ surfaces, but comparable to those of H‐terminated diamond with various surface orientations.^[^ ^41^ ^]^

**Table 1:** Experimentally measured energy levels of the 0.25 ML Sc‐adsorbed oxidized SCD (100) surface at various stages of the sample preparation. Note: electron affinity (χ) values are determined from the results of the UPS analysis.

0.25 ML Sc‐adsorbed *C*_d_ (100)	*E*_F_–*E*_VBM_ [eV]		ϕ [eV]		χ [eV]
	XPS	UPS^a^ ^)^	UPS	EF‐PEEM	
As‐deposited	1.32	1.25	5.25	5.34	1.03
300 °C	1.38	1.33	5.42	5.53	1.28
500 °C	1.12	1.03	4.18	4.27	−0.26
700 °C	0.90	0.82	3.63	3.70	−1.02
900 °C	0.84	0.78	3.80	3.88	−0.89
Air exposure	0.85	0.80	3.73	3.79	−0.94

^a)^ The values are indicated with a negative sign, as the Fermi level is set to zero.

In addition, the “thermally activated” ScO‐diamond surface was not only stable against exposure to ambient conditions but also showed a considerable improvement in electronic properties. Specifically, the work function was lowered to 3.79 eV, while the EA became slightly negative (−0.94 eV) compared to the value after the final annealing point. This can be linked to the adsorption of additional O atoms to compensate for those lost during annealing at 900 °C in order to create a strong surface dipole (see later).

To elucidate the changes observed in the full‐scale UPS spectra (Figure 3a), it is essential to consider the energy‐level diagrams for the ScO‐diamond surface at the particular step. While the electronic states of each step can differ depending on the positions of energy levels (e.g., E_F_ and the vacuum level, *E*_vac_), two specific cases are demonstrated for following the Sc deposition at room temperature and exposure to air, as shown in Figure 3d,e, respectively. Given that the doping level of the sample is identical to that of Sc‐terminated bare diamond,^[^ ^41^ ^]^ the bulk Fermi level of the *p*‐type doped sample is pinned at 0.29 eV above the VBM, as described in the method of Maier et al.^[^ ^51^ ^]^ Moreover, knowing the difference between *E*_F_ and *E*_VBM_ from Figure 3b for each step of the sample preparation and considering the downward bending nature of the bands, we infer that the band bending varies considerably, ranging from 0.96 eV for the as‐deposited step to 0.51 eV for the air‐exposure step, exhibiting the transition from PEA to NEA, as reported elsewhere.^[^ ^52^ ^]^ For the as‐deposited sample, the wide inelastic spectrum is suddenly cut‐off at *E*_vac_, which is significantly above the CBM by an energy difference of 1.03 eV, thus hindering electron emission (Figure 3d). In contrast, the surface, “activated thermally” and subsequently exposed to air, shows a signal intensity spanning as far as 0.94 eV below the CBM before reaching zero at *E*_vac_, as shown in Figure 3e. Therefore, electron emission below the CBM predominantly arises from within the bandgap regions, attributed to surface emissions facilitated by evanescent waves at the metal–vacuum interface.^[^ ^53^ ^]^ We note that the CBM position in this study is determined using the method proposed by Graupner et al.,^[^ ^54^ ^]^ which allows greater reliability in identifying the SE peak maxima associated with CBM emission compared to other approaches.^[^ ^55^, ^56^ ^]^ Meanwhile, using the difference between *E*_F_ and *E*_VBM_ from Figure 3b and the work function value (ϕ) from Figure 3c, the EA can thus be calculated from

(2) $$\chi =\phi +\left(E_{F}-E_{\mathrm{VBM}}\right)-E_{g}$$

where *E*_g_ is the experimental bandgap of diamond of 5.47 eV. Experimentally measured values of EA and work function for each step of the sample preparation are listed in Table 1. For comparison, the most negative EA and work function values measured for other metal adsorbates on the oxygenated surface are summarized in **Table** **2**.

**Table 2:** The most negative electron affinities (χ) with corresponding work function values (ϕ) of different metal adsorbates on the pre‐oxidized diamond (100) surface. Note: metals like Cu, Co, and Zr were not included due to exhibiting PEA.

Termination	Coverage [ML]	ϕ [eV]	χ [eV]	Refs.
Li	1	4.0	<0	[23]
Mg	0.5	2.6	−2.0	[24]
Al	1	4.3	−0.3	[25]
Ti	0.5	4.5	−0.9	[30]
Sc	0.25	3.63	−1.02	this study

A careful inspection of electron emission from a particular surface requires an understanding of the surface dipole, which in turn governs the charge transfer and binding mechanism between the Sc adsorbate atom and the oxidized diamond surface. To support this, DFT calculations were conducted to analyze the distribution of surface charge density for the lowest‐energy configuration of ScO‐terminated diamond (recall Figure 1e), with detailed findings provided in the Supporting Information. According to Hirshfeld charge analysis, 0.21 electrons per Å^−3^ are transferred from the oxidized diamond surface, accumulating around the Sc atom. As a result, the dipole is aligned along the O─Sc bond, with a substantial positive charge, localized on the Sc atom, facing outward toward the vacuum (see Figure S6, Supporting Information). This is in excellent agreement with a complex “dipole model” consisting of one negatively charged (δ^−^) atom sitting between two positively charged (δ^+^) atoms, wherein outward‐oriented dipoles act to lower the electrostatic barrier for electron emission.^[^ ^37^, ^57^, ^58^ ^]^ As illustrated in Figure 3f and Figure S6c (Supporting Information), O attracts electrons from the surface C and Sc atoms, making itself more negative, while the C and Sc atoms become more positive, creating a strong outward‐facing dipole. The presence of Sc is crucial, as it not only acts as an adsorbate atom but also enhances the binding and/or stability of the surface, thereby reducing O desorption due to the high affinity of Sc toward O. Thus, from a fundamental perspective, this elucidates the mechanisms behind surface‐dipole formation as a means to lower the work function and extend the cathode lifetime, as previously demonstrated in various Sc‐based thermionic cathodes.^[^ ^37^, ^59^ ^]^

To expand our investigation, we now utilize EF‐PEEM to map the surface in real space. Although conventional UPS is a well‐established technique, the presence of an additional peak stemming from secondary‐electron emission complicates the direct determination of the work function for NEA surfaces. As a result, alternative surface‐science techniques, such as PEEM, are preferred for their ability to provide more precise measurements, thanks to their superior spatial resolution and enhanced surface sensitivity.^[^ ^60^ ^]^

**Figure 4:**
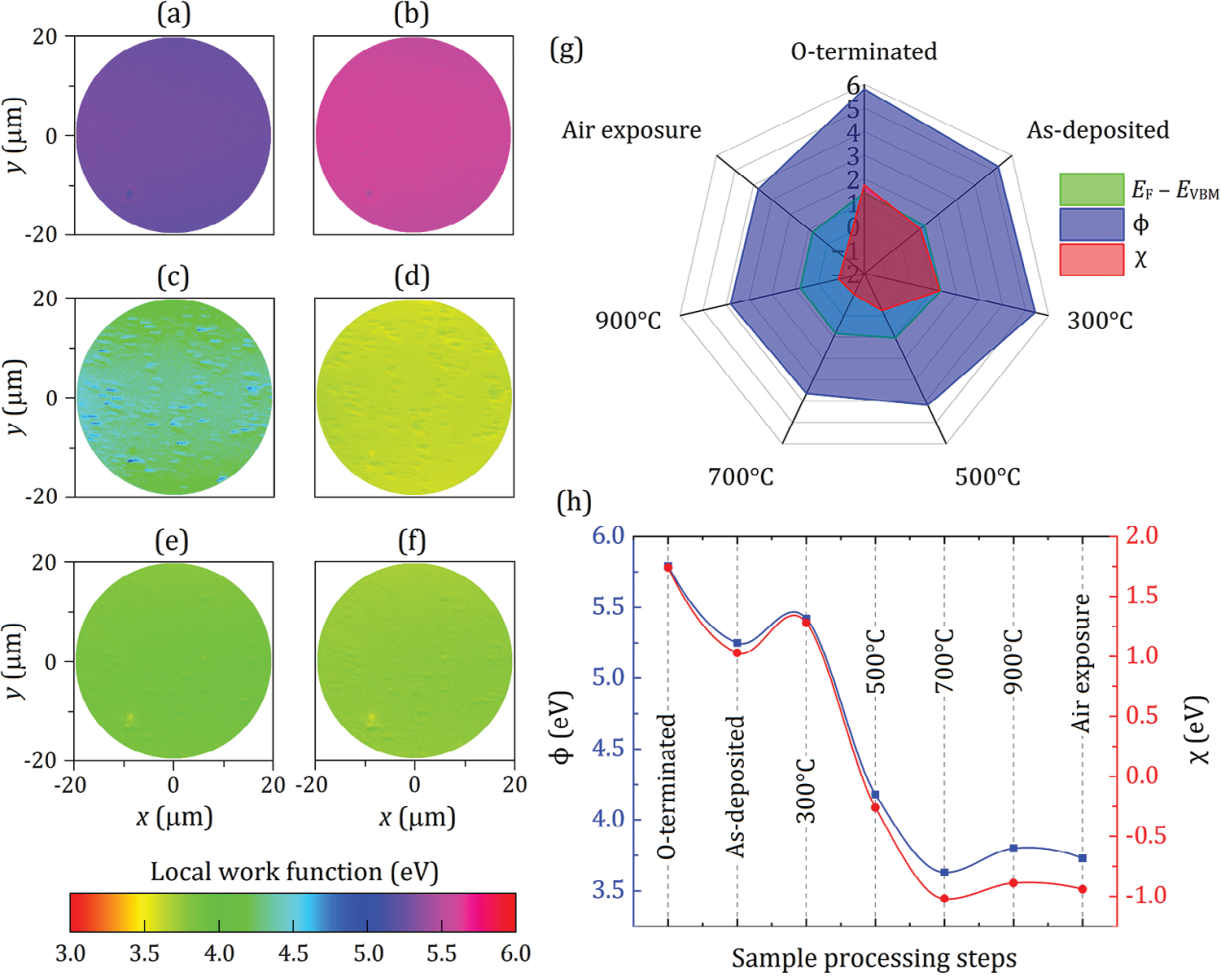
a–f) Color‐coded LWF maps (a) after 0.25 ML Sc deposition at room temperature, and subsequent annealing at b) 300 °C, c) 500 °C, d) 700 °C, and e) 900 °C on the oxygenated SCD (100) sample, and (f) following exposure under ambient conditions. The field of view for each map is 37.5 µm. g) The radar plot depicts a dataset of energy values measured using a region‐selected UPS technique, as referenced in Table 1. h) Variations of work function (ϕ) and (χ) values as a function of sample processing steps (i.e., deposition, annealing, and air exposure). Work function and EA values of the O‐terminated diamond (100) surface are provided as a reference for the starting point. The red and blue curves serve as visual guides.

**Figure** **4**a–f presents color‐coded local‐work‐function (LWF) maps illustrating the electronic terrain for the Sc‐adsorbed oxidized diamond (100) sample, and depicting variations resulting from Sc deposition, thermal annealing, and air exposure. The work function values were determined by averaging across the single‐crystal surface of interest, utilizing data obtained from PEEM full‐wavevector imaging, and are listed in Table 1. These work function values showed significant variation, ranging from 3.63 to 5.42 eV, throughout the processes of Sc deposition, successive in vacuo annealing and exposure to ambient conditions. Moreover, the LWF maps displayed a high level of uniformity across the surface, with no noticeable micron‐sized spots or defects over an area of diameter 37.5 µm. This level of uniformity is a marked improvement compared to the somewhat patchy coverage previously reported with adsorbates like Al and Ti on the oxygenated diamond (100) surface.^[^ ^25^, ^30^ ^]^

Although DFT simulations indicated that “island formation” was thermodynamically unlikely for the Sc─O─C_d_ system,^[^ ^31^ ^]^ annealing at 500 °C led to the appearance of 2D islands or 3D clusters of Sc atoms on the surface, which had a relatively higher work function, as seen in Figure 4c. This is likely a result of a variable amount of unbonded Sc atoms deposited on the oxidized surface, creating a sub‐ML coverage. With further increases in temperature, the surface quality considerably improved and gave lower LWF values, as shown in Figure 4d,e. Particularly, after annealing at 700 °C, 2D islands of Sc remained on the surface with reduced LWF values, consistent with the dominant part of the surface (Figure 4d). The highest annealing temperature (i.e., 900 °C) further smoothed the islands after desorption of a sufficient amount of O atoms (see previous sections), leading to a very uniform surface without obvious submicron‐sized blemishes (Figure 4e). Finally, the surface quality further exhibited stability versus exposure to atmospheric conditions, with the LWF value of the ScO‐terminated diamond (100) surface only slightly reduced after air exposure compared to that after annealing at 900 °C.

To enhance the reliability and validity of the results, work function values and the energy difference of *E*_F_–*E*_VBM_ were determined using two different techniques (UPS and PEEM) at various stages of the sample preparation process. Using the relevant inputs obtained from the UPS analysis, the calculated EA magnitudes taken from Table 1, along with the work function and *E*_F_–*E*_VBM_ values, are visually presented in Figure 4g as a function of sample processing. To better understand the numerical trend during the sample preparation steps, changes in work function and EA values are illustrated in Figure 4h. The results show that the trend for both energy values is consistent up to 500 °C annealing, whereas starting from 700 °C, there is a small deviation likely due to the electronic structure changes discussed earlier.

## 3 Conclusions

We have reported a detailed study into the electronic and electron‐emission properties of preoxidized (100) diamond surfaces following deposition of a 0.25 ML of scandium metal and then annealing at various temperatures. A range of surface‐science techniques was used to characterize this Sc─O‐diamond surface, in particular, to determine the EA and work function values associated with different preparation conditions. Changes in the surface state resulting in a strong dipole are confirmed by photoelectron spectroscopy techniques (XPS and UPS) as a function of the annealing temperature. Furthermore, a dipole model favorable for electron‐emission enhancement is proposed by complementary ab initio DFT calculations, highlighting the significance of scandium as an adsorbate atom. Following deposition of a 0.25 ML of Sc and annealing up to 900 °C, the surface exhibits significant resilience against exposure to ambient conditions, unequivocally yielding a robust NEA surface of −0.94 eV, with a corresponding work function value of 3.79 eV, some of the best values ever recorded for a metal‐O‐diamond system. Importantly, these values do not significantly deteriorate at temperatures in vacuo up to 700 °C.

We therefore suggest that ScO‐terminated diamond, with its temperature‐ and air‐stable NEA surface, is perhaps the best candidate yet reported for a wide range of advanced materials applications, particularly those that rely on efficient electron emission from a surface into a vacuum. These examples include thermionic emitters, secondary electron emission devices, dynode detectors, field‐effect transistors (FETs), X‐ray sources, betavoltaic batteries, gammavoltaic cells, and more.

## 4 Experimental Section

### Sample Preparation

Square‐shaped undoped SCD (100) plates (product code: 145‐500‐0549) with an average surface roughness (*R*_a_) better than 2 nm, obtained from Element Six, Ltd (Ascot, UK), were used for all experiments in this study (see Figure S7, Supporting Information). The pristine substrates were cleaned in a boiling acid mixture (6.5 g KNO_3_ in 100 mL of 95% H_2_SO_4_) for 3 h to remove residual contaminants resulting from the polishing process. A conductive 500‐nm‐thick BDD capping layer was grown homoepitaxially onto the clean SCD (100) substrates via a hot‐filament chemical vapor deposition (HF‐CVD) technique to prevent surface charging during subsequent photoemission measurements.^[^ ^25^, ^30^, ^41^ ^]^ During the HF‐CVD process, a gas mixture containing 1% CH_4_ and 5% B_2_H_6_ (a B:C ratio of 812.5 parts per million (ppm)) in H_2_ was employed, with a total flow rate of 202.1 standard cubic centimeters per minute (sccm). This process resulted in a boron concentration in the layer of ≈10^20^ cm^−3^, as previously confirmed by secondary ion mass spectrometry (SIMS).^[^ ^61^ ^]^ To achieve an atomically flat surface, the BDD‐capped substrates underwent treatment in a microwave plasma‐enhanced CVD (MPE‐CVD) reactor, utilizing a pure H_2_ plasma through a multi‐step process, as reported in the previous studies.^[^ ^39^, ^41^ ^]^ Freshly H‐terminated samples were then immediately oxidized by a UV‐ozone treatment process, as detailed in ref. [29] which fully replaced all the H atoms on the diamond surface with O, in various bonding configurations (as discussed in section 1).

The oxidized SCD (100) sample served as the starting point benchmark for this study and was introduced into a NanoESCA II surface‐science analysis system under ultra‐high vacuum (UHV) conditions. The sample was initially annealed at 300 °C for 1 h to eliminate residual contaminants and was then transferred between the deposition, analysis, and annealing chambers, remaining in situ in UHV for all subsequent experiments.

For air‐exposure procedures, following a 900 °C anneal the ScO‐terminated SCD (100) samples were left in ambient laboratory conditions for 14 days, then reloaded into a UHV chamber, annealed at 300 °C for 1 h, and subsequently re‐measured using XPS, UPS, and EF‐PEEM.

### Surface‐Science Analysis

The surface structure of SCD at any particular stage of the sample preparation for the oxidized and ScO‐terminated SCD (100) samples was examined using SPA‐LEED measurements at an electron energy of 130 eV. The objectives were to verify: i) the reciprocal‐lattice structure and surface reconstruction after the Sc deposition and annealing procedures, ii) the intensity of surface domains (e.g., (1 × 1) or (2 × 1)) in reciprocal space, and iii) qualitatively determine the presence of adsorbate atoms of interest, specifically O and Sc for the oxidized and ScO‐terminated surfaces, respectively. To aid in the analysis of the experimental data, the reciprocal‐space patterns for each surface were simulated using the *LEEDpat4* software.^[^ ^62^ ^]^

Core‐level XPS analysis was performed using a monochromatic Al Kα source (1486.7 eV) with a total energy resolution of 600 meV at pass energies of 20 and 50 eV for all high‐resolution and survey spectra, respectively. A Scienta Omicron Argus analyzer was positioned at an electron polar angle of 25° to the surface normal. A polycrystalline gold film was employed to calibrate the XPS signals by aligning the Au 4f_7/2_ peak to 84.0 eV. To determine the relative concentrations of component elements, the atomic cross‐sections for emission lines were obtained from ref. [63] The stoichiometry between the two elements was calculated by taking the ratio of their background‐subtracted emission peak areas, divided by their respective emission‐line cross‐sections. The XPS peaks were analyzed utilizing *XPSPEAK41* software (version 4.1) and verified using the well‐known XPS fitting program *CasaXPS* (version 2.3.18),^[^ ^64^ ^]^ ensuring reproducible Gaussian–Lorentzian line profiles with a Shirley background,^[^ ^65^ ^]^ as reported earlier in the studies.^[^ ^29^, ^41^ ^]^ The detection of only C 1*s*, O 1*s*, and Sc 2*p* signals on the surface is indicative of a successful cleaning process and the absence of contamination (recall Figure 2).

An EF‐PEEM analyzer was employed to perform region‐selected UPS and PEEM full‐wavevector imaging, using a monochromatic He (I) discharge lamp (21.22 eV) and a nonmonochromatic Hg‐vapor lamp (*hν* < 5.8 eV), respectively, as low‐energy UV‐light sources at room temperature. For PEEM imaging, the field of view value was set to ≈37.5 µm by iris tuning, with a maximum lateral resolution of 20 nm. A 2D map of real‐space work‐function acquisitions was then constructed by selecting EF‐PEEM images pixel‐by‐pixel in a particular area of interest. For each pixel of the image, the photoemission threshold (*I*) is derived from:^[^ ^66^ ^]^

(3) $$I=\frac{I_{\max}}{2}\mathrm{erfc}\left(\frac{\phi -\left(E-E_{F}\right)}{\sigma \sqrt{2}}\right)+I_{\mathrm{off}}$$

where *E*_F_ is the Fermi energy, *I*_max_ is the maximum intensity of each pixel, *I*_off_ is the intensity offset, ϕ is the LWF, and 𝜎 is the standard deviation of the Gaussian distribution due to energy broadening.

### Scandium Deposition

Scandium was deposited from a Sc rod (99.99%) purchased from Testbourne Ltd (Hampshire, UK), using an e‐beam evaporator operating at 8.8 W and 1 kV at room temperature to achieve 0.25 ML coverage, as detailed earlier in ref. [41] The background pressure was 1 × 10^−9^ mbar, rising to 5 × 10^−9^ mbar during deposition. The deposition rate was monitored using a quartz‐crystal microbalance, and the amount of evaporated Sc was precisely determined by the area of the core‐level Sc 2*p* peak measured via XPS.

### Annealing Process

A coiled W filament embedded just behind the sample holder in the preparation chamber's manipulator was used as a direct current resistive heating element, with a maximum power of ≈130 W. To reach certain temperature points of 300, 500, 700, and 900 °C, the current to the heater was calibrated, ranging between 2.25 and 7.15 A. The annealing time at each temperature point was 15 min.

### DFT Calculations

A first‐principles simulation was conducted using the Cambridge Serial Total Energy Package (CASTEP) code.^[^ ^67^ ^]^ Electronic properties of the oxidized and ScO‐terminated (100) diamond surfaces were calculated employing the generalized gradient approximation (GGA) with the Perdew–Burke–Ernzerhof (PBE) functional. To construct the electron density, plane waves with an energy cut‐off of 800 eV as the basis set, as optimized in the previous DFT works, were utilized.^[^ ^31^, ^41^ ^]^ A 14‐carbon‐layer‐thick diamond slab with unit‐cell dimensions of 3 × 2 was used to simulate the surface reconstruction and relaxation of the two different terminated (100) surfaces in this study. To match the boron‐doping level observed in the experimental study (i.e., the B : C ratio of 812.5 ppm or 0.3%), one carbon atom in the second layer of the slab was substitutionally replaced with a boron atom, as detailed earlier.^[^ ^41^, ^68^ ^]^

## Conflict of Interest

The authors declare no conflict of interest.

## Supporting information

Supporting Information is available from the Wiley Online Library or from the author.

## Data Availability

The data that support the findings of this study are available from the corresponding author upon reasonable request.
