# Supplementary material for: Enhanced Electron Emission Performance and Air‐Surface Stability in ScO‐Terminated Diamond for Thermionic Energy Converters
Source: Small. 2024 Sep 2;20(48):2405408. doi: 10.1002/smll.202405408 (PMC11600692; doi:10.1002/smll.202405408)
Supplement: Supplementary file 1 — Supporting Information is available from the Wiley Online Library or from the author. [file SMLL-20-2405408-s001.pdf]

NANO · MICRO
small

Supporting Information

for *Small*, DOI 10.1002/smll.202405408

Enhanced Electron Emission Performance and Air-Surface Stability in ScO-Terminated Diamond for Thermionic Energy Converters

Ramiz Zulkharnay, Neil A. Fox and Paul W. May*

Supporting Information for

Enhanced Electron Emission Performance and Air-Surface Stability in ScO-terminated Diamond for Thermionic Energy Converters

Ramiz Zulkharnay[a*], Neil Fox[a,b] and Paul W. May[a]

[a] *School of Chemistry, University of Bristol, Cantock's Close, Bristol, BS8 1TS, UK*

[b] *School of Physics, H.H. Wills Physics Laboratory, University of Bristol, Tyndall Avenue, Bristol, BS8 1TL, UK*

*Corresponding author: E-mail: ramiz.zulkharnay@bristol.ac.uk

SI-1 Oxidized diamond (100) surface

SI-1.1 UV-ozone treatment

Surface oxidation of single-crystal diamond (SCD) (100) was performed *via* UV-ozone treatment due to the favorable surface configurations for binding with Sc atoms.[1] The principle of UV-ozone treatment is the surface oxidation process with atomic oxygen during the formation or dissociation of O_3 molecules. The oxidation procedure was conducted using a UVO-cleaner kit (Model 42, Jelight Company Inc.) at room temperature and pressure. A mercury vapor lamp situated ~4 cm above the samples creates UV irradiation of atmospheric oxygen with a wavelength of 184.9 nm to form O_3 which decomposes to $O^{\bullet}$ and O_2. The reactive atomic O reacts with the diamond surface, replacing the C_d–H bonds with a variety of oxygen-containing surface groups[1] producing a full monolayer (ML) coverage of O within 25 min.

SI-1.2 SPA-LEED measurements

SPA-LEED was performed on the UV-ozone-treated sample to assess the quality and structure of the oxidized diamond (100) surface. As expected, the oxidized single-crystal diamond (SCD) exhibits a distinctive (1 × 1) LEED pattern, consisting of a single 90°-rotated domain in a square lattice,[2-3] as seen in Figure S1a. The UV-ozone oxidation technique led to a highly ordered surface structure which manifested as sharp (1 × 1) domains and minimal

background intensity, surpassing the LEED patterns observed using other oxidation techniques.[1] The findings from the LEED study were in good agreement with EF-PEEM imaging (see section SI-1.4 later).

The experimental SPA-LEED results for the oxygenated diamond (100) sample were confirmed by the *LEEDpat* software simulation, resulting in predictions of real and reciprocal-space patterns. The (1 × 1) structure for the O-terminated diamond was constructed using a single-domain matrix ($M = \begin{bmatrix} 1 & 0 \\ 0 & 1 \end{bmatrix}$) rotated by 90°. The reciprocal unit-cell vectors represent the two O adatoms. Meanwhile, the primitive unit-cell lattice is square, as expected for the O-terminated diamond (100) with a (1 × 1) reconstructed surface, as shown in Figures S1a and b, respectively.

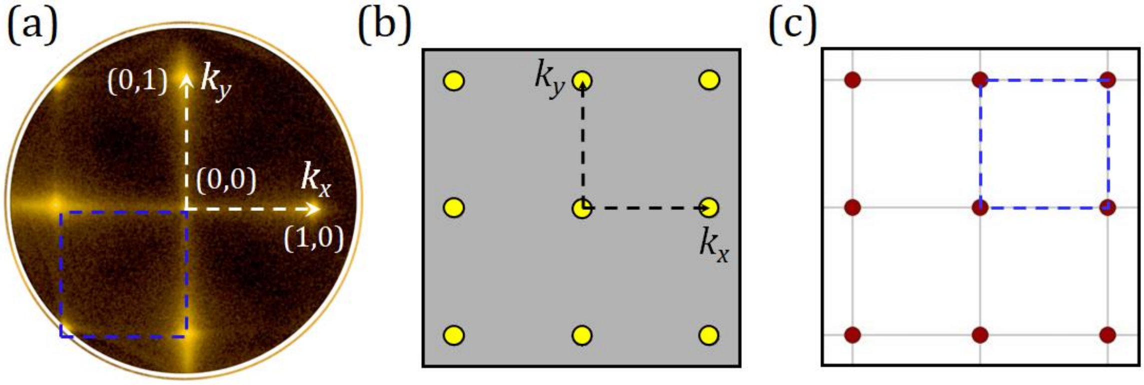

Figure S1. Real-space and reciprocal-space patterns of oxidized SCD (100). (a) Experimental LEED pattern of the UV-ozone oxidized sample obtained at a beam energy of 130 eV. White dashed arrows indicate the primitive reciprocal-lattice vectors along the k_x and k_y axes, while the blue dashed square corresponds to a (1 × 1) diffraction symmetry. Drawings of a superposition of the simulated reciprocal- and real-space patterns for C(1 × 1)-(100):O are illustrated in panels (b) and (c), respectively. In panel (b), yellow circles refer to the (1 × 1) domains in a square lattice, while black dashed arrows are the primitive reciprocal-lattice vectors showing (1 × 1) diffraction spots. In panel (b), the red circles indicate the position of individual domains, whilst the blue dashed square shows the (1 × 1) geometry.

SI-1.3 XPS analysis

The deconvoluted C 1*s* peak is typically used to characterize the nature of surface bonding in oxidized diamond and is resolved into four main components, in agreement with previous studies.[4-8] Figure 2a displays the prominent peaks observed in the C 1*s* spectra for the oxidized diamond (100) surface, which vary depending on the oxidation method used. Regardless of how the surface is terminated, the prominent peak in each spectrum remains unaltered. This peak (labelled C1) has a binding energy (BE) of ~285.2 eV and is assigned to bulk sp^3-hybridized C_d atoms. Due to the oxidized surface, a lower intensity peak (C2) at ~283.8 eV is present, attributed to graphitic or amorphous carbon, indicative of sp^2 C=C groups on the surface. For O-terminated diamond, typically three extra peaks can be detected at higher BEs. Significantly, the intensity of these peaks may vary depending on the oxidation techniques employed. The first peak (C3) at 286.6 eV is ascribed to the C_d–O single bonds present in ether (C_d–O–C_d) and hydroxyl (C_d–OH) functionalities. The second and third peaks (grouped together under the label C4) usually arise around ~288 eV and ~289 eV and are associated with the C_d=O double bonds of carbonyl (*i.e.* ketone) and carboxyl (R-COOH) components, respectively, as reported by Ferro *et al*.[9]. In our samples, the carboxyl-group peak is relatively small because the C=O double bonds are primarily due to the ketone component.

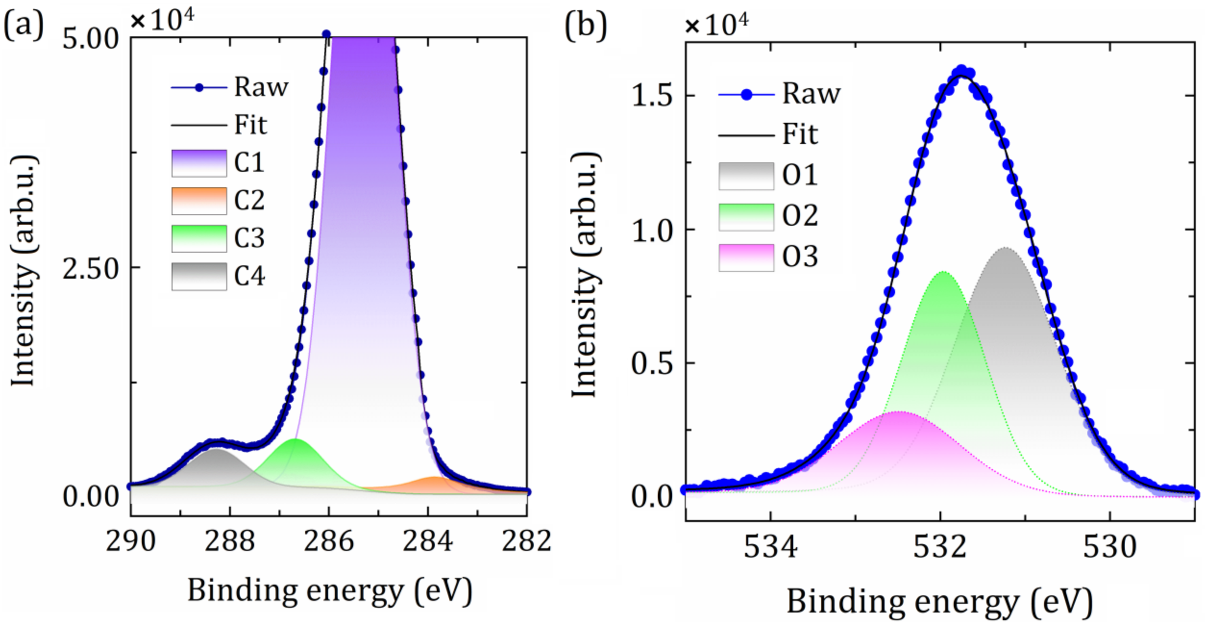

Figure S2. Core-level XPS spectra of (a) C 1*s* and (b) O 1*s* with corresponding peak components for C1-C4 and O1-O3, respectively, (see Table S1) for UV-ozone oxidation. The tops of the C 1*s* peaks are cut off to highlight low-intensity components.

In studies of oxidized single-crystalline and nanocrystalline diamond, the deconvolution of the O 1*s* peak has often revealed two or three distinct components, each attributed to specific factors. However, the findings from the two-component fitting analysis have been contradictory, as demonstrated by the conflicting assignments reported in prior works. For example, Lesiak *et al.*[10] attributed the two components to C_d–OH and C_d=O (ketone) bonds, while others proposed that they originated from C_d–OH and C_d–O–C_d bonds.[11-12] By performing three-component peak fitting, the fitting accuracy for oxygenated ultrananocrystalline and microcrystalline diamond samples was improved, assigning the 3 components to C_d=O, C_d–OH and C_d–O–C_d functional groups.[7,13]

In this study, three-component peak fitting was similarly applied with C_d=O centered at 531.1 eV, C_d–OH around 532 eV, and C_d–O–C_d at ~532.7 eV, labelled as O1, O2 and O3 peaks, respectively, in Figure 2b and Table S1.

Table S1. Spectral fitting attributes (*e.g.* peak assignment and BE) of the C 1*s* and O 1*s* peaks for the UV-ozone treated sample. *All* XPS spectra assignments are taken from Figures S2a and b for C 1*s* and O 1*s*, respectively.

**Spectra attributes**		**UV-ozone**
C 1*s*		
**Peak**	**Assignment**	**BE / eV**
C1	Bulk sp^3 C_d–C_d	285.3
C2	Surface sp^2 C=C	283.9
C3	C_d–O	286.7
C4	C_d=O	288.3
O 1*s*		
O1	C_d=O	531.1
O2	C_d–OH	531.9
O3	C_d–O–C_d	532.6

SI-1.4 UPS and EF-PEEM measurements

Figure S3a presents the energy distribution of secondary electrons for the UV-ozone oxidized (100) SCD sample, as determined by region-selected UPS analysis. This method also verifies the oxidation state of the sample. The oxidation of the H-terminated surface significantly reduces the intensity of the NEA peak at higher BEs, as indicated by a comparison of the UPS spectra of the oxygenated surface in Figure S3a with those of the H-terminated

diamond (100) sample from our previous study.[14] In the UV-ozone-treated sample, the high cut-off energy is shifted to 15.41 eV, resulting in a kinetic-energy value that exceeds the band gap, characteristic of a surface with positive electron affinity (PEA).[15] Furthermore, intense valence band (VB) emissions appeared at ~3.7 eV and 8.5 eV contributing to O 2*p* states [16]. The intensity of these bands depends on the oxidation method used.[1]

Meanwhile, the color-coded map of the oxidized SCD sample (Figure S3b) shows some inhomogeneity with micron-sized blemishes appearing across the surface, with the local work function (LWF) value of ~5.9 ± 0.1 eV, in good agreement with those from other works.[1,17] The EF-PEEM imaging data are consistent with the findings from the SPA-LEED study discussed earlier (see section SI-1.2).

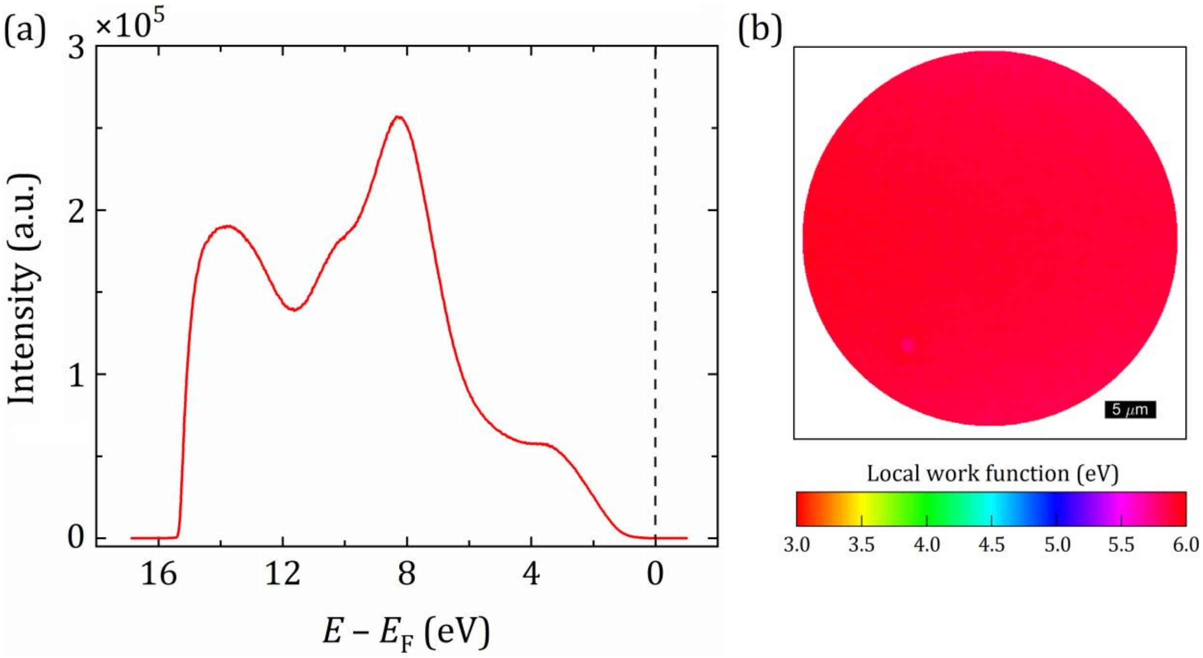

Figure S3. (a) Region-selected UPS spectra of the O-terminated diamond (100) surface. The dashed vertical line indicates the Fermi level which is set at zero. (b) Color-coded LWF map of the oxidized diamond. The field of view for each map is 37.5 μm.

SI-2 ScO-terminated diamond (100) surface

SI-2.1 XPS analysis

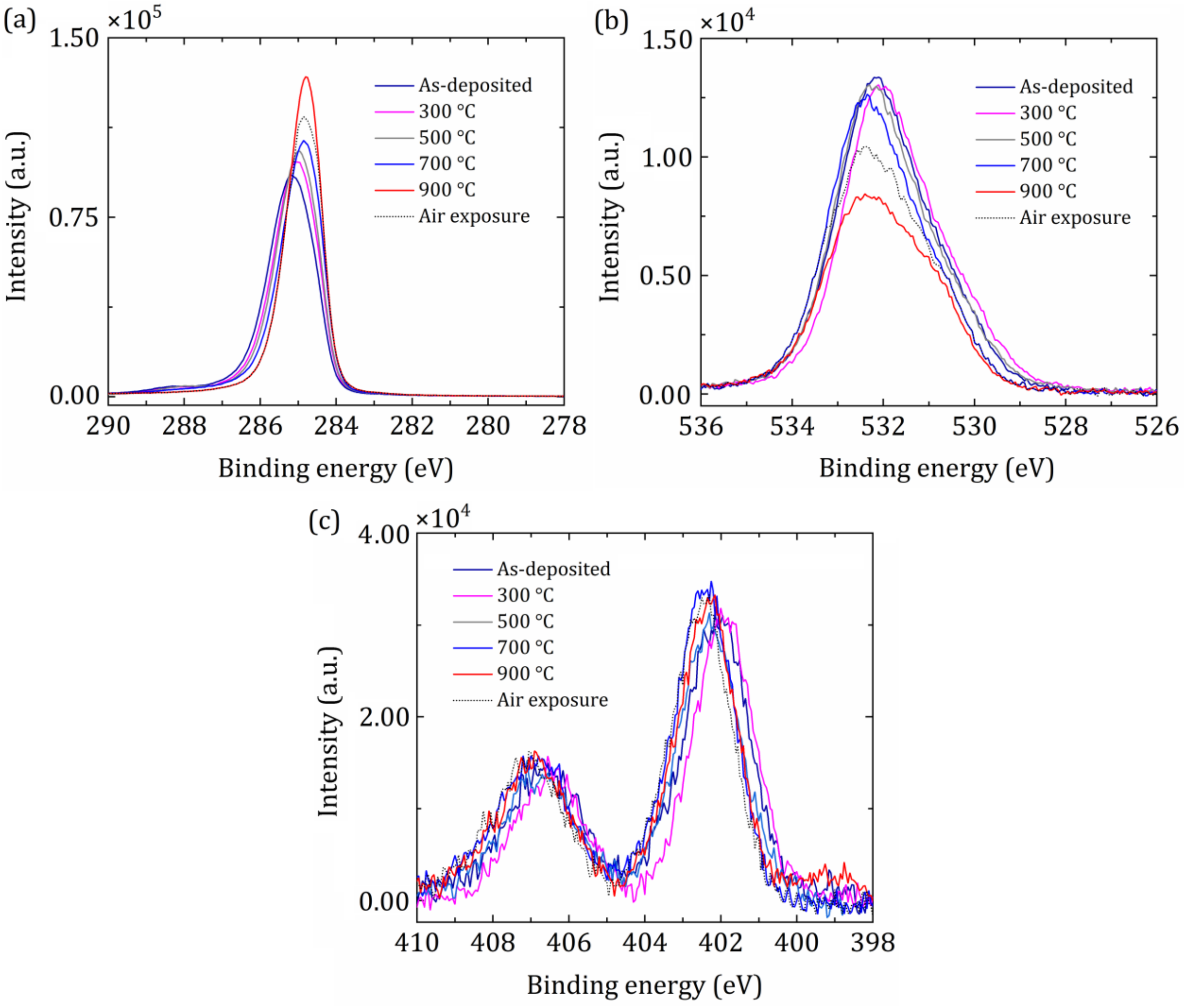

Figure S4. Raw XPS spectra of (a) C 1*s*, (b) O 1*s* and (c) Sc 2*p* peaks for the ScO-terminated SCD (100) surface at various steps of the sample preparation, including Sc deposition at room temperature, *in vacuo* annealing up to 900 °C and exposure under ambient conditions. 'As-deposited' means 0.25 ML Sc deposition at room temperature.

SI-2.1 DFT modelling

SI-2.2 Convergence testing

To determine the optimum simulation time and accuracy, preliminary testing simulations using the main computational parameters, such as the cut-off energy and the number of *k*-points, were conducted to gain an understanding of the impact of these parameters on the total energy (or enthalpy) of a hydrogenated BDD (100) slab within geometry optimisation. Variation in the total energy and computing time with the cut-off energy and the number of *k*-

points for the *x* and *y* directions, are shown in Figure S5. Due to the shape of the unit cell in reciprocal space, the *k*-points for Brillouin-zone sampling were chosen to be parallel to the (100)-oriented surface of the diamond slab. By increasing the cut-off energy and the number of *k*-points, the total energy was stabilized, meaning the system converged. As a result, the most favorable value of 800 eV was determined as the cut-off energy, while the optimum number of *k*-points was 18 (or Monkhorst-Pack grid size of $6 \times 6 \times 1$). To ensure that DFT modelling of the surface terminations of interest had also converged, further calculations were performed for the oxidized diamond (100) and 0.25 ML of Sc on the oxidized surface.

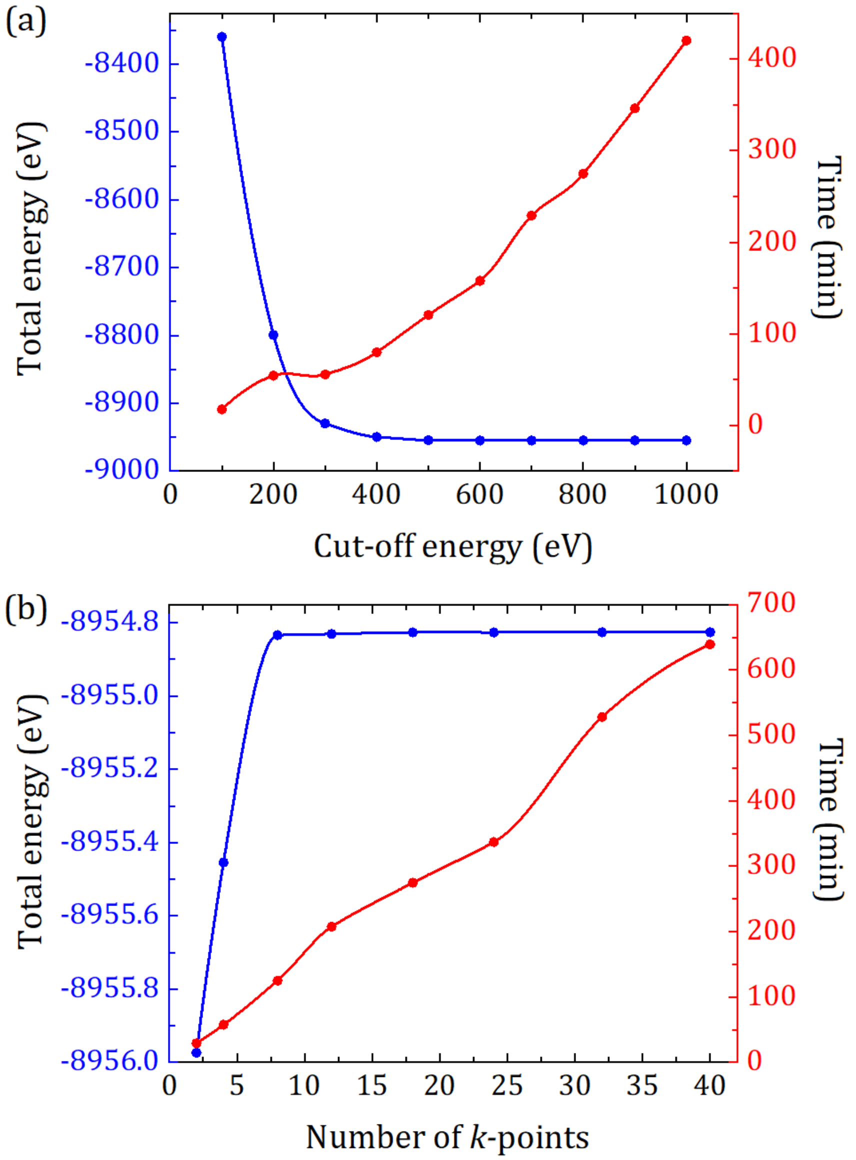

Figure S5. Total energy and simulation time changes upon (a) the cut-off energy and (b) the number of *k*-points used for a hydrogenated BDD (100) slab within the geometry optimisation process.

SI-2.3 Calculation of the distribution of surface charge density

Detailed surface-charge-distribution analysis was performed for the lowest-energy structure of the ScO-terminated diamond (100) surface. Figure S6 shows the electrostatic-potential variation (upper panel) coupled with the charge-density map (middle panel) and the charge-density-difference plot (lower panel), along the *z*-axis of the three terminations. These plots were used to examine the changes to the work function (or the EA) of the oxidized diamond surface with the addition of 0.25 ML Sc. Because of the extremely low charge density in the vacuum gap, the exchange-correlation potential has been disregarded, as in a previous study.[18] The calculation of ionisation energy, I, or the EA is obtained from a simple subtraction of energy values (Equations 1 and 2), and so the accuracy of the predicted values is not strongly affected by the exchange-correlation potential.

The ionisation-energy calculation for each surface is based on the energy difference between the vacuum and the valence-band maximum (VBM).

$$I = E_{\mathrm{vac}} - E_{\mathrm{VBM}} = (E_{\mathrm{vac}} - V_{\mathrm{av.slab}}) - (E_{\mathrm{VBM,bulk}} - V_{\mathrm{av.bulk}}) \qquad (1)$$

E_{vac} is the energy of the vacuum level which is set to zero, E_{VBM} is the energy of the VBM, while $V_{\mathrm{av.slab}}$ and $V_{\mathrm{av.bulk}}$ are the average electrostatic potential energies in the slab and bulk, respectively. $E_{\mathrm{VBM,bulk}}$ is the VBM calculated for bulk diamond. The difference between $E_{\mathrm{VBM,bulk}}$ and $E_{\mathrm{av.bulk}}$ is equal to 10.52 eV which has already been computed.[19] The values for E_{vac} and $V_{\mathrm{av.bulk}}$ are extracted from the CASTEP binary check file using the *pot1d* CASTEP tool. This makes use of the macroscopic average method of Fall *et al.*[20]

Due to the well-known underestimation of the band gap of diamond by GGA, the ‘scissor correction’ is applied to adjust the calculated findings to be consistent with experimental values.[18] Thus, the EA value, χ, is calculated from the ionisation energy by subtracting the experimental band gap (E_{g} 5.47 eV):

$$\chi = I - E_{\mathrm{g}} \qquad (2)$$

The work function (ϕ) refers to the lowest energy required to emit electrons from the solid surface and can be determined as follows:

$$\phi = E_{\mathrm{vac}} - E_{\mathrm{F}} \qquad (3)$$

where E_{F} is the Fermi energy.

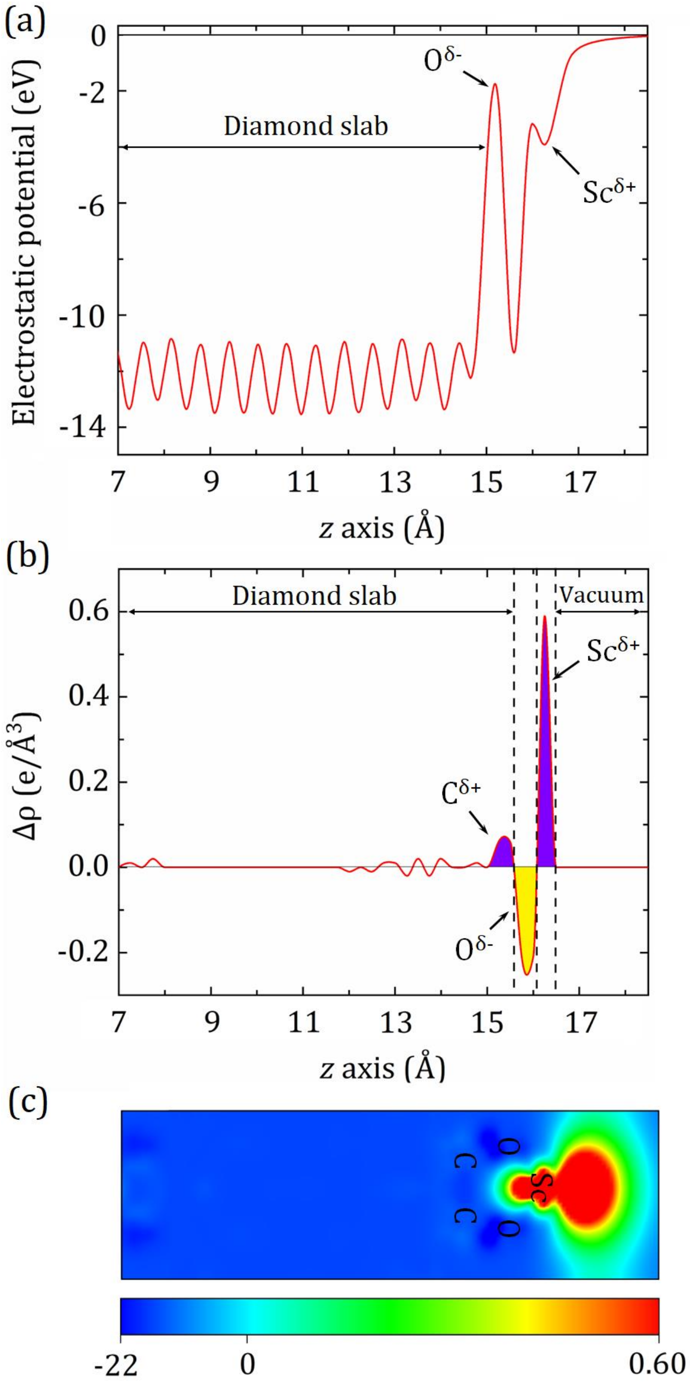

Figure S6. (a) Plane-averaged electrostatic potential (upper plot) with corresponding (b) charge-density map (middle panel) and (c) charge-density-difference plot (lower panel) for the 0.25 ML Sc-terminated oxidized diamond (100) surface, along the *z*-direction. In the upper plot, E_{vac} is set to zero (shown by the upper black line), whilst the average potential of the bulk C atoms inside the slab, $V_{av.slab}$ lies typically between ~–13 eV and –11 eV. The positions of maximum negative and positive charges are indicated by $O^{\delta-}$ and $Sc^{\delta+}$, respectively, in the surface dipoles. In the middle plot, the purple and yellow regions depict electron accumulation and depletion, respectively. In the lower plot, the surface structure is displayed on a color-coded background representing the charge density value at that position.

The surface atoms (C and O) and adsorbate species (Sc) are indicated by the corresponding positions within the slab configuration. To express the EA calculation easily with Equations 1 and 2, the vacuum level is set to zero, while the average electrostatic-potential energies in the slab oscillate between ∼–13 eV and –11 eV. At the surface, the potential is heavily modulated due to the surface-charge distribution caused by the Sc adsorbate. The potential curves at the surface provide the local charge densities, which satisfy Poisson's equation:

$$\nabla^2 V = -\frac{\rho}{\varepsilon} \tag{4}$$

where V is the electrostatic potential energy per unit cell charge, ρ the total charge density, and ε the permittivity. Using the current sign convention, the peaks indicate positions of higher local negative-charge density ($O^{\delta-}$), whilst the troughs correspond to higher local positive-charge density ($Sc^{\delta+}$), as shown in the upper plot of Figure 6a. In the lower plots, the surface-charge-distribution features are consistent with the corresponding charge-density difference and color-coded charge-density map (Figure 6b and c, respectively).

As expected for the structure with more negative EA, the electrostatic potential drop at z = ∼15.6 Å from the ScO-terminated surface is considerably higher than those observed for Sc adsorbed onto bare and nitrogenated diamond (100) surfaces.[21] This behavior is similar to that observed for the H-terminated diamond surface, indicating large NEA values.[22] The dipole-moment features are also consistent with the higher positively charged region observed in the charge-density map of 0.25 ML Sc on the oxygenated diamond surface (Figure S6c). Furthermore, the dipole is oriented along the O–Sc bonds, with the larger positive charge located on the Sc atoms facing the vacuum. This makes the ScO-terminated surface practical for further use as a thermionic cathode, as discussed in the main paper.

SI-3 Single-crystal diamond (100)

SI-3.1 Surface roughness measurement

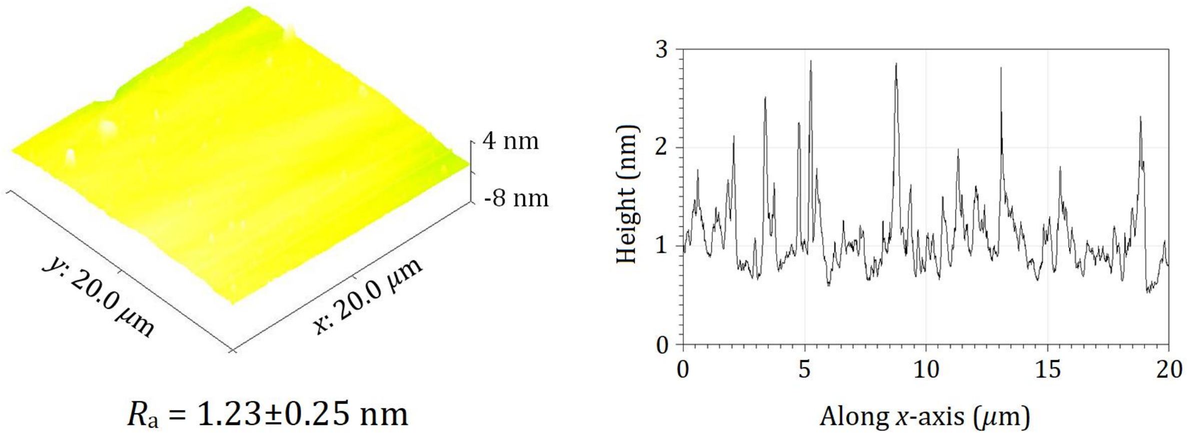

Figure S7. Atomic Force Microscopy (AFM) 3D map with corresponding surface roughness plot for as-received single-crystal diamond (100) sample.

References

[1] R. Zulkharnay, G. Zulpukarova, P. W. May, *Appl. Surf. Sci.* **2024**, 658, 159776.

[2] P. E. Pehrsson, T. W. Mercer, *Surf. Sci.* **2000**, 460, 49–66.

[3] G. Wan, M. Cattelan, N. A. Fox, *J. Phys. Chem. C* **2019**, 123, 4168–4177.

[4] F. Klauser, S. Ghodbane, R. Boukherroub, S. Szunerits, D. Steinmüller-Nethl, E. Bertel, N. Memmel, *Diam. Relat. Mater.* **2010**, 19, 474–478.

[5] S. Ghodbane, D. Ballutaud, A. Deneuville, C. Baron, *Physica Status Solidi A – Applications and Materials Science* **2006**, 203, 3147–3151.

[6] S. Torrengo, R. Canteri, R. Dell'Anna, L. Minati, A. Pasquarelli, G. Speranza, *Appl. Surf. Sci.* **2013**, 276, 101–111.

[7] C. Li, X. Zhang, E. F. Oliveira, A. B. Puthirath, M. R. Neupane, J. D. Weil, A. G. Birdwell, T. G. Ivanov, S. Kong, T. Gray, H. Kannan, A. Biswas, R. Vajtai, D. S. Galvao, P. M. Ajayan, *Carbon* **2021**, 182, 725–734.

[8] G. Alba, M. P. Villar, R. Alcantara, J. Navas, D. Araujo, *Nanomaterials* **2020**, 10.

[9] S. Ferro, M. Dal Colle, A. De Battisti, *Carbon* **2005**, 43, 1191–1203.

[10] B. Lesiak, L. Kövér, J. Tóth, J. Zemek, P. Jiricek, A. Kromka, N. Rangam, *Appl. Surf. Sci.* **2018**, 452, 223–231.

[11] A. Gaisinskaya, R. Akhvlediani, R. Edrei, E. Alagem, Z. Joselzon, A. Hoffman, *Diam. Relat. Mater.* **2010**, 19, 305–313.

[12] J. Navas, D. Araujo, J. C. Piñero, A. Sánchez-Coronilla, E. Blanco, P. Villar, R. Alcántara, J. Montserrat, M. Florentin, D. Eon, J. Pernot, *Appl. Surf. Sci.* **2018**, 433, 408–418.

[13] K. Huang, X. Hu, H. Xu, Y. Shen, A. Khomich, *Appl. Surf. Sci.* **2014**, 317, 11–18.

[14] R. Zulkharnay, P. W. May, *J. Mater. Chem. A* **2023**, 11, 13432–13445.

[15] G. Speranza, S. Torrengo, A. Miotello, L. Minati, I. Bernagozzi, M. Ferrari, M. Dipalo, E. Kohn, *Diam. Relat. Mater.* **2011**, 20, 560–563.

[16] K. P. Loh, X. N. Xie, S. W. Yang, J. C. Zheng, *J. Phys. Chem. B* **2002**, 106, 5230–5240.

[17] M. C. James, M. Cattelan, N. A. Fox, R. F. Silva, R. M. Silva, P. W. May, *Phys. Status Solidi B* **2021**, 258, 2100027.

[18] K. M. O'Donnell, T. L. Martin, N. L. Allan, *Chem. Mater.* **2015**, 27, 1306–1315.

[19] K. M. O'Donnell, T. L. Martin, N. A. Fox, D. Cherns, *Phys. Rev. B* **2010**, 82, 115303.

[20] C. J. Fall, N. Binggeli, A. Baldereschi, *J. Phys.: Condens. Matter* **1999**, 11, 2689–2696.

[21] R. Zulkharnay, N. L. Allan, P. W. May, *Carbon* **2022**, 196, 176–185.

[22] M. C. James, F. Fogarty, R. Zulkharnay, N. A. Fox, P. W. May, *Carbon* **2021**, 171, 532–550.
